# Effects of Climate Change on the Global Distribution of *Trachypteris picta* (Coleoptera: Buprestidae)

**DOI:** 10.3390/insects16080802

**Published:** 2025-08-02

**Authors:** Huafeng Liu, Shuangyi Wang, Yunchun Li, Shuangmei Ding, Aimin Shi, Ding Yang, Zhonghua Wei

**Affiliations:** 1 The Key Laboratory of Southwest China Wildlife Resources Conservation of the Ministry of Education, College of Life Sciences, China West Normal University, Nanchong 637009, China; hero876@126.com (H.L.); liyc2260@cwnu.edu.cn (Y.L.); aiminshi2003@126.com (A.S.); 2 Student Affairs Department, Sichuan University of Arts and Science, Dazhou 635000, China; wsy_ws1@163.com; 3 The Institute of Scientific and Technical Research on Archives, National Archives Administration of China, Beijing 100050, China; shuangmeiding@163.com; 4 State Key Laboratory of Green Pesticides, Guizhou University, Guiyang 550025, China; 5 State Key Laboratory of Agricultural and Forestry Biosecurity, MARA Key Lab of Surveillance and Management for Plant Quarantine Pests, College of Plant Protection, China Agricultural University, Beijing 100193, China

**Keywords:** jewel beetle, MaxEnt, pest, suitable area, *Trachypteris picta*

## Abstract

*Trachypteris picta* (Pallas, 1773) is a significant pest that can cause serious damage to poplars and willows. To assess the impact of climate change on the suitable habitats of *T. picta*, this study conducted a comparative analysis of its global suitable habitats using climatic factors, global land use type, and global vegetation from different periods, in combination with the maximum entropy (MaxEnt) model. The results indicate that the annual mean temperature (Bio01), mean temperature of the coldest quarter (Bio11), precipitation of the coldest quarter (Bio19), and isothermality (Bio03) are the four most important climate variables determining the distribution of *T. picta*. Under the current climate conditions, the highly suitable areas are primarily located in southern Europe, covering an area of 2.22 × 10^6^ km^2^. Under future climate scenarios, the suitable habitat for *T. picta* is expected to expand and shift towards higher latitudes. In the 2050s, the SSP5-8.5 scenario has the largest suitable area compared to other scenarios, while the SSP2-4.5 scenario has the largest suitable area in the 2090s. In addition, the centroids of the total suitable areas are expected to shift toward higher latitudes under future climate conditions. The results of this study provide valuable data for the monitoring, control, and management of this pest.

## Simple Summary

Global climate change has significantly affected species distribution. Understanding the impact of climate change on the distribution of forest pests is essential for effective monitoring and control. *Trachypteris picta* (Pallas, 1773) is an important forest pest in the family Buprestidae. To explore the impact of climate change on the distribution of *T. picta*, we simulated suitable habitat under the current conditions and three climate change scenarios (SSP1-2.6, SSP2-4.5, and SSP5-8.5) for the 2050s and 2090s using the maximum entropy (MaxEnt) model. The predicted results suggest that the total area of suitable habitats is expected to increase and shift towards higher latitudes under future climate conditions. The expansion of suitable areas was primarily located in southern Europe, with smaller, fragmented expansion areas concentrated in Northwest China under future climate scenarios.

## 1. Introduction

The family Buprestidae, commonly known as jewel beetles, is a significant group within the order Coleoptera, comprising over 15,000 known species worldwide [1]. Some buprestid beetles are renowned for their striking appearance and are collected as ornamental items. However, certain borer species, such as *Chrysobothris succedanea* Saunders, 1873 [2], *Agrilus zanthoxylumi* Li, 1989 [3], *Agrilus mali* Matsumura, 1924 [4], and *Agrilus planipennis* Fairmaire, 1888 [5,6], are notorious for causing substantial economic damage to agriculture and forestry during outbreaks.

The genus *Trachypteris* is a member of the family Buprestidae, with its only known species, *Trachypteris picta* (Pallas, 1773), mainly distributed across the Nearctic, Oriental, and Palaearctic realms [1]. The species exhibits considerable color variation, with some individuals displaying colorless elytra, while others possess five pairs of spots on the elytra. This variability contributes to the existence of multiple synonyms for this species [7]. The primary host plants of *T. picta* are species within the genera *Populus* and *Salix* [8,9,10]. The larvae of this species are borers that overwinter in the larval stage, and the adults primarily emerge in June and July in China [11]. Over the past three decades, *T. picta* have caused significant economic damage to forestry in the northwest region of China due to their outbreaks, and they are regarded as important forestry pests [8,12,13]. The forestry department attaches great importance to monitoring the risk of pest spread; however, the impact of climate change on the distribution of this pest remains unclear.

Climate change events have been a consistent feature throughout the evolutionary history of the Earth, with most extant species surviving through specific climatic cycles [14,15]. However, studies indicate that the rate of future temperature changes will exceed that of the warming observed during the Pleistocene–Holocene transition [14,16]. According to the sixth assessment report of the Intergovernmental Panel on Climate Change (IPCC), the global surface temperature in 2020 had risen by 1.09 °C compared to the pre-industrial levels (1850–1900). Climate change has significantly impacted the distribution of insects [17,18,19,20].

Currently, the impact of climate change on species’ suitable habitats is primarily studied using species distribution models (SDMs), also known as ecological niche models. Several SDMs have been proposed, including the CLIMEX [21,22,23], maximum entropy modeling (MaxEnt) [24], ecological niche factor analysis (ENFA) [25], Genetic Algorithm for Rule Set Production (GARP) [26], Enphylo [27], and bioclimate analysis and prediction system (BIOCLIM) [28]. Among these, MaxEnt is widely used for predicting species distribution of animals [20,29,30,31,32,33] and plants [34,35] due to its high predictive accuracy and stability.

Some studies have shown that changes in climate conditions significantly impact the distribution ranges of species [36,37,38]. However, due to the differences in ecological habits among various species, their adaptations to climate change vary, and there is no consensus on the effects of global warming on insect distribution. While some studies suggest that rising temperatures will gradually increase the suitable habitat area for insects, others indicate that the suitable habitat range may decrease [18,20,36,39,40].

Research on the suitable habitat of buprestid beetles in relation to climate change is limited [41,42,43]. To investigate the changes in the suitable habitats of *T. picta* under climate change, this study employed MaxEnt in conjunction with ArcGIS, using 21 bioclimate factors, to conduct a comparative analysis of their potential distribution. The results of this study provide valuable data for the future monitoring and management of *T. picta*.

## 2. Materials and Methods

### 2.1. Obtaining and Processing Occurrence Data

**Figure 1:**
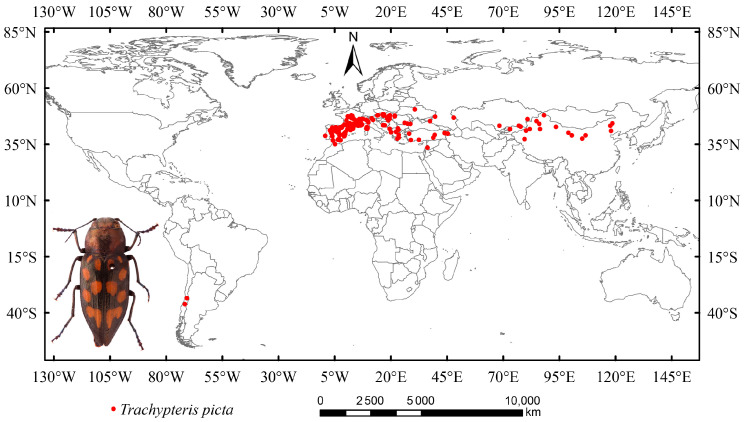
The global distribution and habitus of *Trachypteris picta*.

In this study, occurrence data for *T. picta* were collected from the National Animal Collection Resource Center (http://museum.ioz.ac.cn/, accessed on 18 June 2025), the China National Knowledge Infrastructure (CNKI, https://www.cnki.net/, accessed on 18 June 2025), and the Global Biodiversity Information Facility (GBIF, https://www.gbif.org/, accessed on 18 June 2025). Data that recorded only states or provinces (without latitude and longitude) were excluded, as a recent study indicated that vague data can impact the accuracy of species distribution models [44]. A total of 462 distribution points were initially obtained (Figure 1). Duplicate occurrence data were removed using Excel. Secondary sampling was performed on a 2.5-arc grid to reduce sampling bias and mitigate overfitting, while also addressing spatial autocorrelation effects [45]. After cleaning and filtering, 269 distribution points for *T. picta* remained and were used for modeling tests.

### 2.2. Environmental Variables

**Figure 2:**
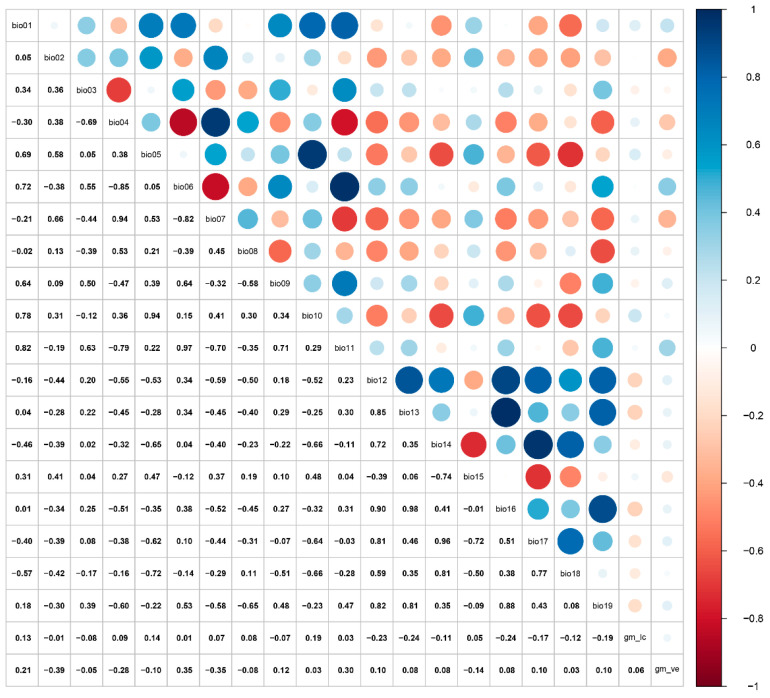
Pearson correlation analysis of environmental variables.

At a large spatial scale, bioclimatic and soil variables are critical factors influencing species distribution models [46]. For this study, climatic variables at a 2.5 arc-minute spatial resolution were obtained from WorldClim (http://www.worldclim.org/, accessed on 18 June 2025). The global land cover type v3 and vegetation (percent tree cover) v2 were downloaded from GitHub (https://globalmaps.github.io/, accessed on 18 June 2025) [47]. For the global land cover type, the data type, data source, and resolution were bytes (16 bits), MODIS data 2013 (Terra and Aqua, National Aeronautics and Space Administration, USA), or 15 arcseconds, respectively. For global vegetation, the data type, data source, and resolution are bytes (8 bits), MODIS data 2008 (Terra and Aqua), and 15 arcseconds, respectively. The environmental variables encompass both current (1970–2000) and future climate projections (2041–2060, 2081–2100). The current climate data span the period 1970–2000, while future projections were derived from the BCC-CSM2-MR model under the Coupled Model Intercomparison Project Phase 6 (CMIP6) [48,49]. Three shared socioeconomic pathways (SSPs) emission scenarios were selected: SSP1-2.6 (low level of greenhouse gas emissions), SSP2-4.5 (intermediate level of greenhouse gas emissions), and SSP5-8.5 (high level of greenhouse gas emissions). Bioclimatic variables were subjected to Pearson correlation analysis to avoid model errors induced by high autocorrelation (Figure 2). The retention criterion was set as |r| > 0.85 for each correlated group, with only the maximally correlated variable being preserved. A final principal component analysis (PCA) transformation was then conducted on the filtered variables. Ultimately, 10 environmental variables were selected to predict the potential suitable habitat (Table 1).

### 2.3. Modeling Methods

The software MaxEnt v3.4.1 and ArcGIS v10.2 were used to construct the global suitable habitat of *Trachypteris picta* in the present study. After the variables were determined, a model calibration was conducted using kuenm in R v4.3.2, setting the regularization multiplier from 0.5 to 4, with an interval of 0.5 each time. In this study, the MaxEnt model was run for 500 iterations with 10,000 background points [50]. In the MaxEnt model, 75% of the species distribution points were used for model construction, with 25% reserved for testing. Model performance was evaluated through 10 replicate runs, with the mean area under the receiver operating characteristic (ROC) curve derived from ensemble predictions [51]. The ROC curve was employed to assess the predictive capability of the MaxEnt model. The area under the curve (AUC) is independent of the threshold value and is widely used to evaluate the accuracy of predictive models [52]. The AUC ranges from 0 to 1, where an AUC < 0.5 signifies model failure, 0.5 < AUC < 0.7 indicates low model accuracy, 0.7 < AUC < 0.8 suggests moderate accuracy, 0.8 < AUC < 0.9 indicates high accuracy, and AUC ≥ 0.9 represents excellent accuracy [24,53]. The model performance was also assessed by the omission rate [54], and model complexity was evaluated by AICc [55]. In ArcGIS v10.2, the Jenks natural breaks classification method was applied to classify and visualize the suitable habitats for *T. picta*. The suitable areas were categorized into four groups: unsuitable area (0–0.133), low suitability area (0.133–0.199), moderate suitability area (0.199–0.438), and high suitability area (0.438–1). The suitable areas were calculated using the SDM Toolbox v2.4 module integrated into ArcGIS.

## 3. Results

### 3.1. Accuracy Evaluation and Dominant Environmental Variables

**Figure 3:**
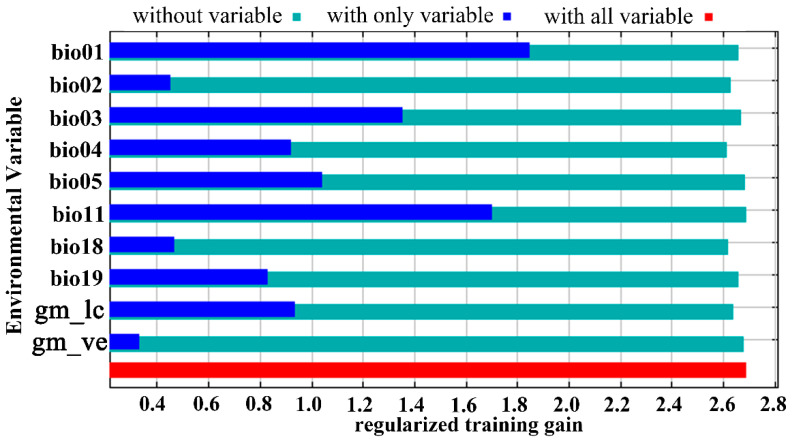
The jackknife test of variables’ importance for *Trachypteris picta* using the MaxEnt model.

In the present study, 269 distribution points of *Trachypteris picta* and 10 environmental variables were used to predict the global suitable habitats under the current and future climate conditions. The AUC value for the training data was 0.980 (Figure S1), which is close to 1.0, indicating excellent model performance. The omission rate at 5% for the MaxEnt model is 0.07, and the lowest value of AICc is approximately 3314.1. The jackknife test showed that the annual mean temperature (Bio01, 26.2%), isothermality (Bio03, 10.3%), mean temperature of the coldest quarter (Bio11, 18.6%), and precipitation of the coldest quarter (Bio19, 14.0%) contributed more significantly than other environmental variables (Table 1; Figure 3). Among the selected 10 environmental variables, the maximum temperature of the warmest month (Bio05, 1.4%) had the smallest contribution. Regarding the permutation importance of bioclimatic variables from the jackknife method, the top three most important variables were annual mean temperature (Bio01, 55.5%), Temperature seasonality (Bio04, 11.6%), and maximum temperature of warmest month (Bio05, 11.5%) (Table 1). These results indicate that the primary environmental variables influencing the geographic distribution of *T. picta* are temperature factors (annual mean temperature, mean temperature of the coldest quarter, isothermality, maximum temperature of the warmest month, and mean temperature of the driest quarter) and precipitation factors (precipitation of the coldest quarter, precipitation of the driest quarter).

The response curve illustrates the relationship between the value of bioclimatic variables and the probability of presence of *Trachypteris picta* (Figure S2). For Bio01, the annual mean temperature, at approximately 13.82 °C, resulted in the highest potential distribution probability of 0.66. For Bio03, the isothermality at around 36.41 produced the highest distribution probability of 0.69. For Bio11, the mean temperature of the coldest quarter, ca. 5.87 °C, yielded the highest potential distribution probability of 0.68. For Bio19, the precipitation of the coldest quarter at around 182.67 mm resulted in the highest distribution probability of 0.72.

### 3.2. Suitable Areas Under the Current Climate Conditions

**Figure 4:**
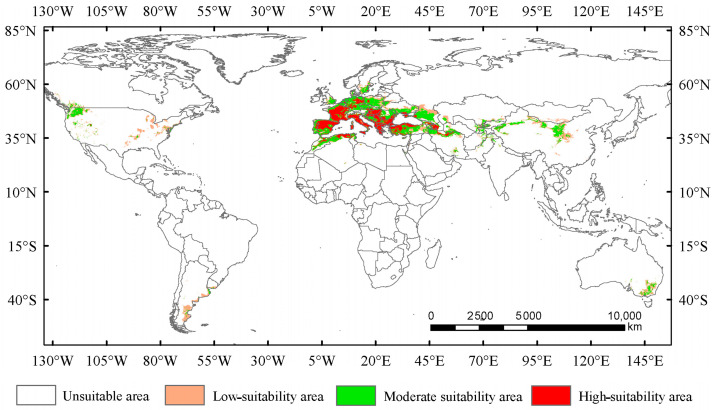
Suitable areas for *Trachypteris picta* under the current climate conditions.

Under the current climate conditions, the total suitable area for *Trachypteris picta* is approximately 7.95 × 10^6^ km^2^ (Figure 4). The predicted suitable habitat area is slightly larger than its actual distribution range. The highly suitable habitat area covers 2.26 × 10^6^ km^2^, accounting for 28.4% of the total suitable area. This area is mainly located in southern Europe (including Portugal, Spain, France, Germany, Italy, Hungary, Greece, Albania, Montenegro, Serbia, and Bulgaria) and northwestern Africa (northern Morocco and Algeria), with fragmented patches of smaller highly suitable areas scattered across parts of Asia (Iran, Azerbaijan, Afghanistan, and Pakistan, also including Xinjiang and Shaanxi provinces of China), southeastern Australia, and the northwestern United States (Figure 4). The moderate-suitability habitat area covers 3.93 × 10^6^ km^2^, accounting for 49.4% of the total suitable area, while the low-suitability habitat area spans 1.75 × 10^6^ km^2^, accounting for 22.1% of the total suitable area.

### 3.3. Change of Suitable Areas Under Future Climate Conditions

**Figure 5:**
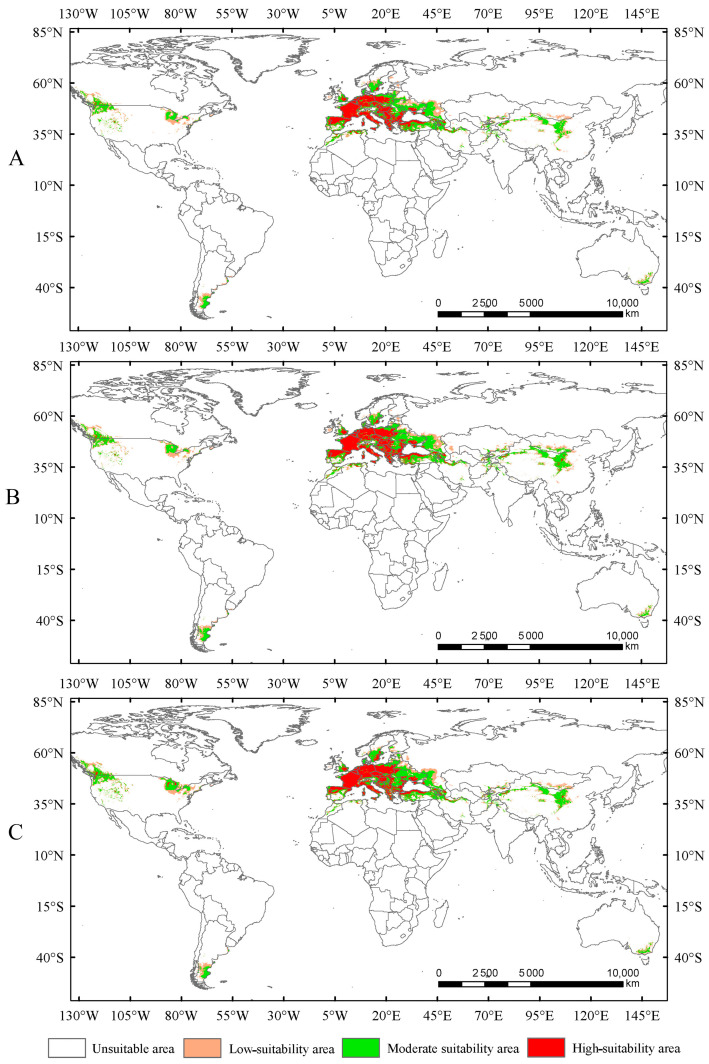
Suitable areas of *Trachypteris picta* under SSP1-2.6 (**A**), SSP2-4.5 (**B**), and SSP5-8.5 (**C**) in the 2050s.

**Figure 6:**
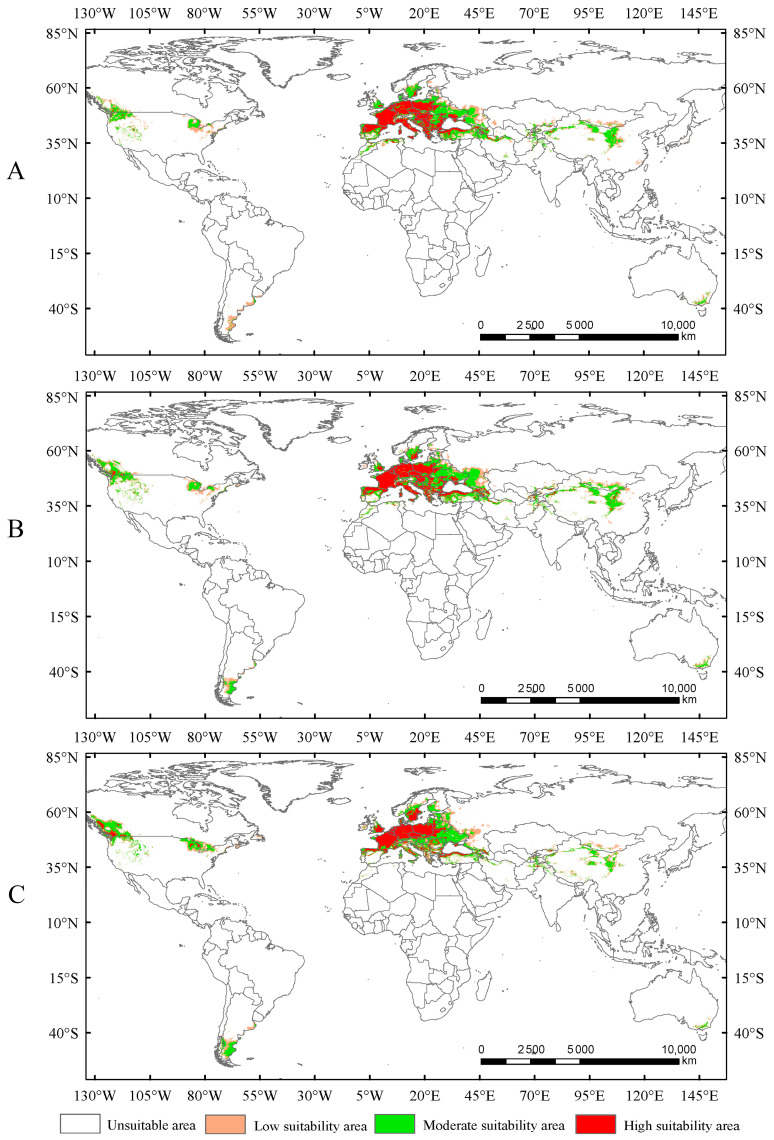
Suitable areas of *Trachypteris picta* under SSP1-2.6 (**A**), SSP2-4.5 (**B**), and SSP5-8.5 (**C**) in the 2090s.

In this study, the potential distribution of *Trachypteris picta* was predicted for the 2050s and 2090s using the optimized MaxEnt model under three different greenhouse gas emission pathways: SSP1-2.6, SSP2-4.5, and SSP5-8.5. During 2041–2060, the suitable habitat areas are 9.01 × 10^6^ km^2^, 9.63 × 10^6^ km^2^, and 9.65 × 10^6^ km^2^ under the SSP1-2.6, SSP2-4.5, and SSP5-8.5 scenarios, respectively, corresponding to increases of 13.40%, 21.16%, and 21.47% compared to the current suitable area (Figure 5). By 2080–2100, the suitable habitat area for *T. picta* is projected to expand by 17.49%, 27.49%, and 18.53% under the three scenarios (Figure 6), respectively, compared to the current suitable habitat. Under scenario SSP2-4.5, during 2080–2100, the total suitable habitat area for *T. picta* demonstrates the highest increase, while the smallest expansion is observed under SSP1-2.6 during 2080–2100, with an increase of 1.39 × 10^6^ km^2^. For highly suitable areas in the 2050s and 2090s, the maximum increase (26.3%) is projected under SSP2-4.5 for 2041–2060, while the minimum increase (4.7%) is expected under SSP5-8.5 for 2080–2100.

### 3.4. Shift of the Centroids Within the Suitable Areas

The centroid refers to the geometric center of a polygon, represented by the average coordinates of the polygon’s vertices. For *Trachypteris picta*, the centroid of the global suitable area is located in the eastern Mediterranean (18.75697° E, 38.05233° N) under the current climate conditions. Under future climate scenarios, the centroids of global suitable areas are expected to shift to the northwest (Figure S3). The migration distance of the centroid is the longest under SSP5-8.5, followed by SSP2-4.5, with the smallest shift under SSP1-2.6. Overall, the increase in temperature causes the centroids of the global suitable areas to shift towards higher latitudes.

## 4. Discussion

Some studies have demonstrated that the impact of climate and soil variables on species distribution is more significant than other factors at a large spatial scale [46,52]. Consequently, this study used climate factors, land use type, and vegetation (tree cover percentage) to explore the global suitable distribution of *Trachypteris picta*. The predicted results from most potential distribution studies align with the current distribution of the species [20,34,35,36], although a few studies do not correspond with the current distribution [56,57]. This discrepancy may arise from the limited number of distribution points in the target area [36,58]. Previous studies have shown that a greater number of species distribution points within a defined range correlates with higher model accuracy [59,60]. However, some scholars also argued that the MaxEnt model is not sensitive to sample size [61]. Recent research has suggested that using imprecise data in species distribution models can lead to inaccurate results [44]. To ensure the accuracy of our findings, distribution points with uncertain location data were excluded from this study. After screening and filtering, 263 distribution points and 10 environmental variables were used for the MaxEnt model. The performance of this model was evaluated using the AUC value, which is 0.980, indicating a high level of accuracy in predicting the suitable habitat for *T. picta* [24,62]. The values of omission rate and model complexity evaluation metric were also suggestive that the model fits the data well.

Bioclimatic factors, especially temperature and rainfall, have an impact on the physiological activities of insects and can also affect their populations and distribution indirectly by restricting their food sources and habitat conditions [63]. Based on the results of the jackknife test, Bio01, Bio03, Bio11, and Bio19 are four key bioclimatic factors that significantly affect the suitable distribution areas of *T. picta*. This supports the above perspective. All insects are poikilotherms, and their body temperature changes with the surrounding environmental temperature, which can affect egg hatching rates and larval development [64]. For *T. picta*, this species overwinters as larvae in the trunks of their host species, with the larvae undergoing diapause from late October to March or April of the following year, while adults appear from April to July [8,12,13]. The temperatures in October and March may play a crucial role in the initiation and termination of diapause. The precipitation in the winter within the distribution area of *T. picta* is snow, which may affect the host and indirectly impact the larvae [41]. As mentioned above, the larvae of *T. picta* inhabit the trunks of their host species and can be transported with the wood, allowing them to spread to other areas and potentially become an invasive species. Therefore, strengthening the inspection of wood from willows and poplars could help prevent the spread of this species. In this study, the predicted potential distribution of *T. picta* is largely consistent with its current distribution. Under the current climate conditions, the highly suitable habitats are primarily concentrated in the southern part of Europe, the northwestern edge of Africa, small scattered areas in China (specifically Xinjiang and Shaanxi), and the northwest of the United States. A small number of suitable habitats for *T. picta* are also identified in southeastern Australia and the northwest of the United Sates; however, no distribution records of *T. picta* have been found in these regions to date. This may be attributed to the fact that the habitats in these regions are similar to those of the existing distribution points, which are characterized by a relatively dry climate [65], and *T. picta* may be present in these regions but has not yet been discovered.

**Figure 7:**
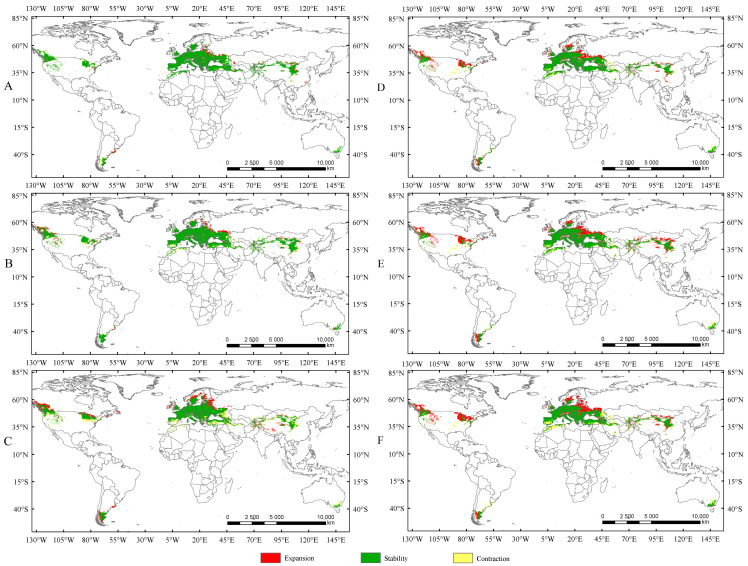
The changes in suitable areas in the 2050s and 2090s compared to those under the current climate conditions. (**A**) Suitable areas of *T. picta* under SSP1-2.6 in the 2050s; (**B**) suitable areas of *T. picta* under SSP2-4.5 in the 2050s; (**C**) suitable areas of *T. picta* under SSP5-8.5 in the 2050s; (**D**) suitable areas of *T. picta* under SSP1-2.6 in the 2090s; (**E**) suitable areas of *T. picta* under SSP2-4.5 in the 2090s; (**F**) suitable areas of *T. picta* under SSP5-8.5 in the 2090s.

Currently, there is no consistent pattern regarding the impact of rising temperatures on the suitable habitat area of insects. The existing studies indicate that under increasing temperatures, the suitable habitat area of some insects may expand [34,40,42], while for others, it may shrink [18,20,39]. In the present study, the suitable habitat areas of *Trachypteris picta* under the current climate conditions are significantly smaller than those projected for future climate scenarios. Compared to the current suitable habitat areas, the suitable habitat areas are expected to expand by 13.4%, 21.16%, and 21.47% under SSP1-2.6, SSP2-4.5, and SSP5-8.5 during 2041–2060, respectively (Figure 7). The suitable habitat areas under SSP1-2.6 and SSP2-4.5 in 2081–2100 are also projected to expand by 3.61% and 5.22%, respectively, compared to those in 2041–2060 (Figure 7), while the suitable area under SSP5-8.5 in the 2050s is expected to be larger than that in the 2090s. This phenomenon is similar to previous studies that demonstrated that rising temperatures can increase the suitable habitat area of insects [31,42]. Under future climate conditions, the expanded areas for *T. picta* primarily extend northward, while a small number of suitable habitats in China expand southward, a trend also observed in other species [18,20,39]. This finding is consistent with most previous studies, which showed that rising temperatures cause the suitable habitats of insects to shift northward [36,42].

## 5. Conclusions

In the present study, the global suitable habitats of *Trachypteris picta* were predicted using the MaxEnt model under the current and future climate conditions. Among the selected environmental variables, the annual mean temperature, mean temperature of the coldest quarter, precipitation of the coldest quarter, and isothermality were found to be more significant than others. Compared to the current distribution, the suitable areas are projected to expand significantly in the periods of 2041–2060 and 2081–2100 under three climate scenarios (SSP1-2.6, SSP2-4.5, and SSP5-8.5). In comparison to the centroid of the current suitable area, the centroids of the suitable areas are shifting towards higher latitudes under future climate scenarios. Based on the suitable areas of *T. picta* under the current climate conditions, the current monitoring activities and pest management mainly focus on the following countries and regions: Portugal, Spain, France, Germany, Italy, Hungary, Greece, Albania, Montenegro, Serbia, Bulgaria, northern Morocco and Algeria, Iran, Azerbaijan, Afghanistan, Pakistan, and Xinjiang and Shaanxi in China. In the future, the pest monitoring areas will expand to include the western border regions of Russia, southern Mongolia, and western China.

## Figures and Tables

**Table 1:** The selected 10 environmental variables used in the MaxEnt model.

Bioclimatic Variable	Description	Contribution (%)	Permutation Importance (%)
Bio01	Annual mean temperature	26.2	55.5
Bio02	Mean daily temperature range	6.2	6.1
Bio03	Isothermality	10.3	5.4
Bio04	Temperature seasonality	7.8	11.6
Bio05	Maximum temperature of the warmest month	1.4	11.5
Bio11	Mean temperature of the coldest quarter	18.6	1.1
Bio18	Precipitation of the warmest quarter	5.8	4.9
Bio19	Precipitation of the coldest quarter	14	2.4
gm_lc	Global land cover type	8.4	1.2
gm_ve	Global vegetation (tree cover percentage)	1.4	0.3

## Data Availability

The original contributions presented in this study are included in the article/Supplementary Material. Further inquiries can be directed to the corresponding authors.

## Supplementary Materials

The following supporting information can be downloaded at: https://www.mdpi.com/article/10.3390/insects16080802/s1, Table S1: Potential suitable area of *Trachypteris picta* under different scenarios; Table S2: Statistical analysis of the changes in the suitable area under different scenarios; Figure S1: Reliability test of the potential distribution for *Trachypteris picta*; Figure S2: Response curves reflecting the relationship between the suitable habitat of *Trachypteris picta* and four key environmental variables; Figure S3: Shift in the geometric center of the suitable area under future climate scenarios.

## Abbreviations

The following abbreviations are used in this manuscript:

*Trachypteris picta*	*T. picta*
Maximum entropy	MaxEnt
Receiver operating characteristic	ROC
Area under the curve	AUC
