# Supplementary material for: Effects of Climate Change on the Global Distribution of Trachypteris picta (Coleoptera: Buprestidae)
Source: Insects. 2025 Aug 2;16(8):802. doi: 10.3390/insects16080802 (PMC12386755; doi:10.3390/insects16080802)
Supplement: Supplementary file 1 [file insects-16-00802-s001.zip › insects-3759672-supplementary.pdf]

Table S1. Potential suitable area of *Trachypteris picta* under different scenarios.

Scenarios, times	Low suitable area (km^2)	Moderate suitable area (km^2)	High suitable area (km^2)
Current	2652668.11	3777694.41	2223396.57
SSP126, 2041–2060	2702017.99	4180757.01	2738170.91
SSP245, 2041–2060	2678877.04	4462842.25	3050966.85
SSP585, 2041–2060	2634846.03	4500552.28	2895470.78
SSP126, 2081–2100	2717758.77	4044532.21	2952837.51
SSP245, 2081–2100	2698857.50	4701806.08	2894129.50
SSP585, 2081–2100	2672494.40	4170936.37	2630991.78

Table S2. Statistical analysis of the changes in the suitable area under different scenarios.

Scenarios, times	Expansion (%)	Absence in both (%)	Stability (%)	Contraction (%)	Expansion (km^2)	Absence in both (km^2)	Stability (km^2)	Contraction (km^2)
current-2050s, SSP126	1.44	91.97	5.68	0.91	1927276.20	122669136.06	7570713.52	1218152.32
current-2050s, SSP245	1.93	91.49	5.59	1.00	2568080.33	122028331.92	7460350.86	1328514.98
current-2050s, SSP585	2.14	91.27	5.17	1.41	2860958.72	121735453.54	6902231.13	1886634.71
2050s-2090s, SSP126	0.46	92.42	6.72	0.40	607829.30	123279459.08	8960273.17	537716.55
2050s-2090s, SSP245	0.64	91.84	6.85	0.66	849617.29	122507229.61	9142974.38	885456.82
2050s-2090s, SSP585	1.13	91.55	5.51	1.80	1505713.51	122116374.74	7356047.33	2407142.52

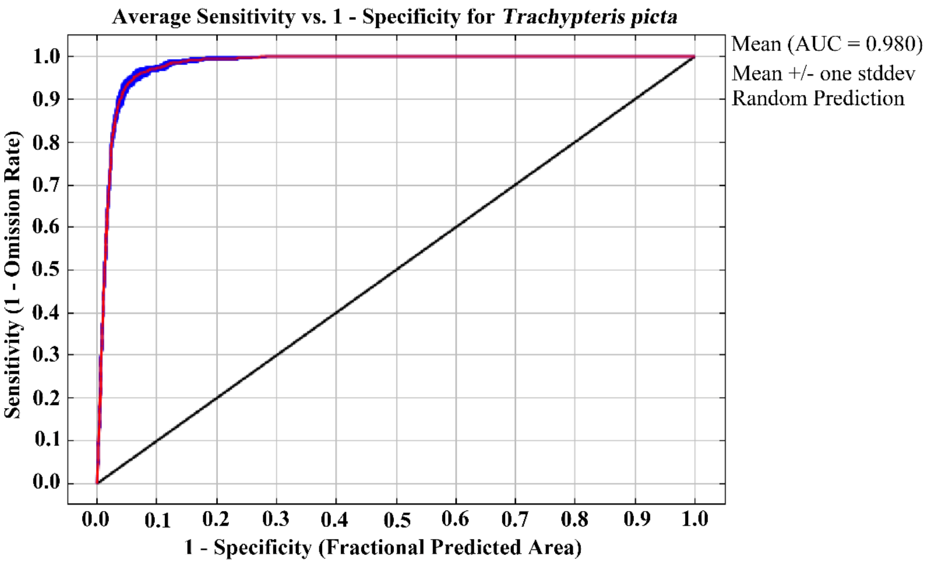

Figure S1. Reliability test of the potential distribution for *Trachypteris picta*.

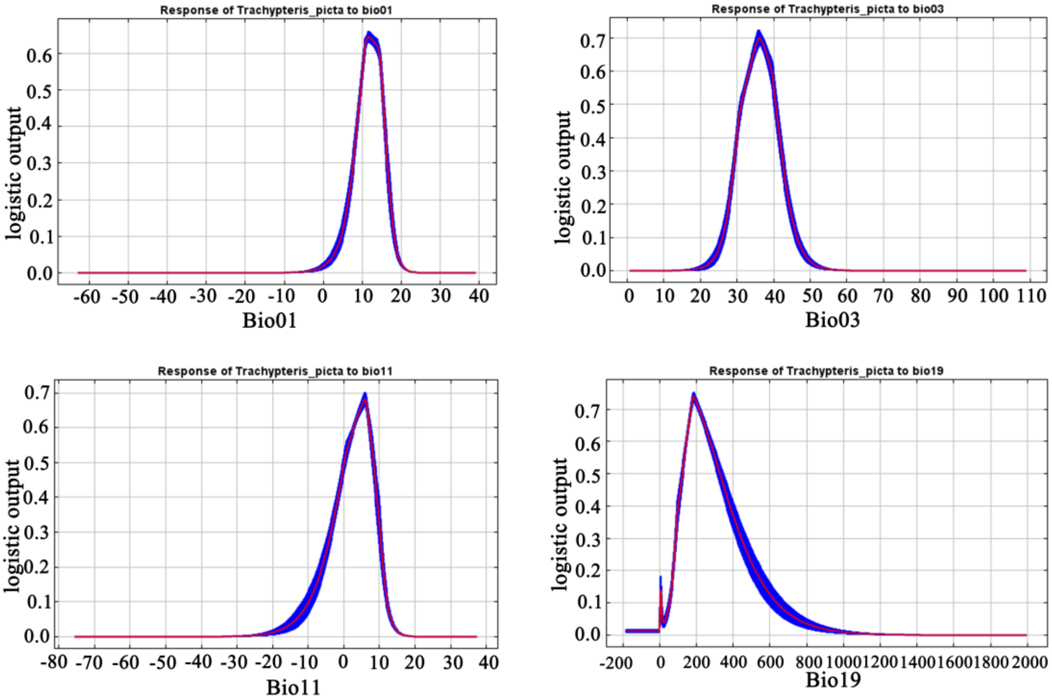

Figure S2. Response curves reflecting the relationship between the suitable habitat of *Trachypteris picta* and four key environmental variables.

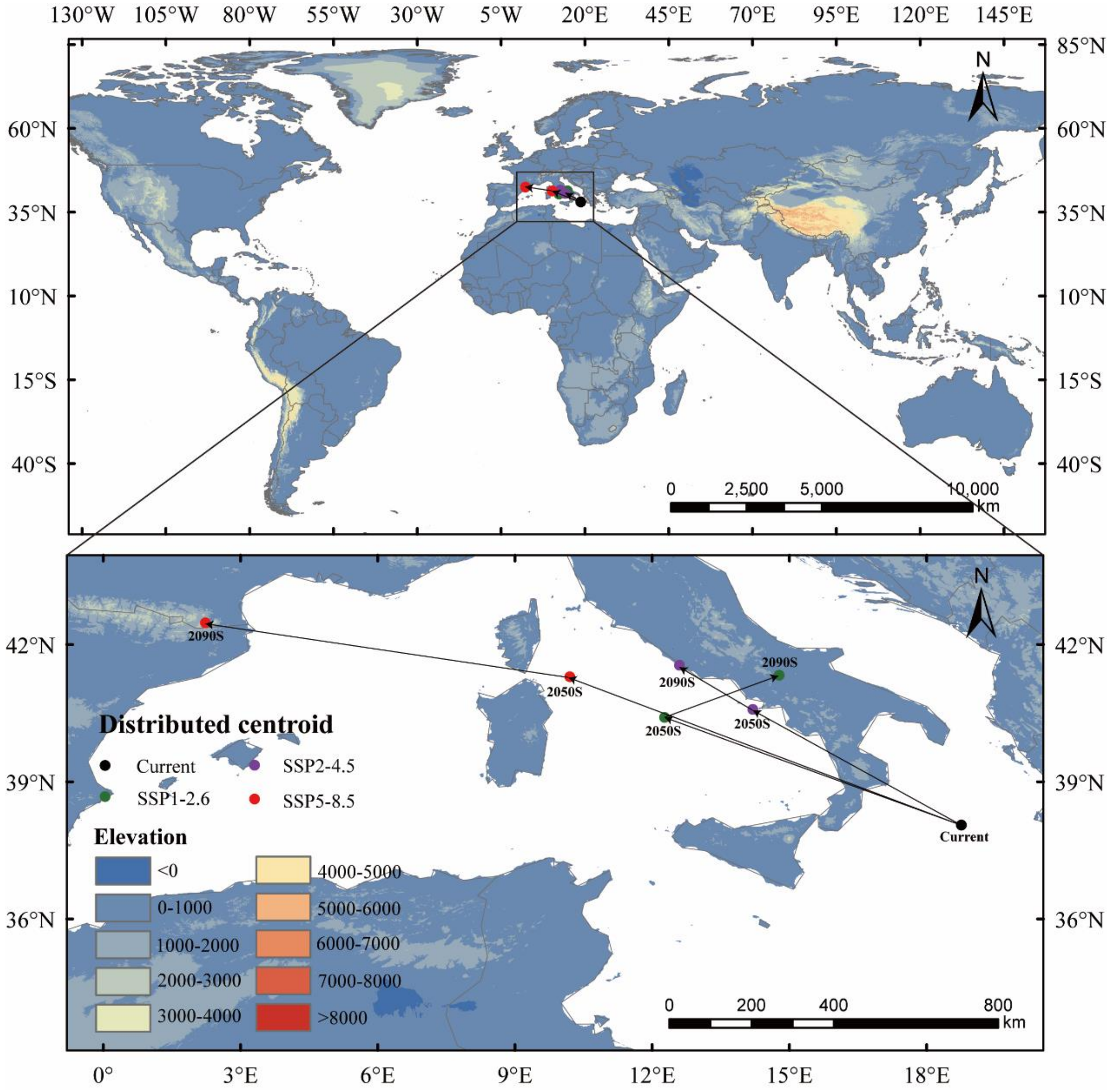

Figure S3. Shift in the geometric center of suitable area under future climate scenarios.
